# Positron Emission Tomography-Based Short-Term Efficacy Evaluation and Prediction in Patients With Non-Small Cell Lung Cancer Treated With Hypo-Fractionated Radiotherapy

**DOI:** 10.3389/fonc.2021.590836

**Published:** 2021-02-25

**Authors:** Yi-Qing Jiang, Qin Gao, Han Chen, Xiang-Xiang Shi, Jing-Bo Wu, Yue Chen, Yan Zhang, Hao-Wen Pang, Sheng Lin

**Affiliations:** ^1^ Department of Oncology, The Affiliated Hospital of Southwest Medical University, Luzhou, China; ^2^ Nuclear Medicine and Molecular Imaging Key Laboratory of Sichuan Province, The Affiliated Hospital of Southwest Medical University, Luzhou, China

**Keywords:** positron emission tomography, radiomics, non-small-cell lung cancer, computed tomography, hypo-fractionated radiotherapy

## Abstract

**Background:**

Positron emission tomography is known to provide more accurate estimates than computed tomography when staging non–small cell lung cancer. The aims of this prospective study were to contrast the short-term efficacy of the two imaging methods while evaluating the effects of hypo-fractionated radiotherapy in non-small cell lung cancer, and to establish a short-term efficacy prediction model based on the radiomics features of positron emission tomography.

**Methods:**

This nonrandomized-controlled trial was conducted from March 2015 to June 2019. Thirty-one lesions of 30 patients underwent the delineation of the regions of interest on positron emission tomography and computed tomography 1 month before, and 3 months after hypo-fractionated radiotherapy. Each patient was evaluated for the differences in local objective response rate between the two images. The Kaplan Meier method was used to analyze the local objective response and subsequent survival duration of the two imaging methods. The 3D Slicer was used to extract the radiomics features based on positron emission tomography. Least absolute shrinkage and selection operator regression was used to eliminate redundant features, and logistic regression analysis was used to develop the curative-effect-predicting model, which was displayed through a radiomics nomogram. Receiver operating characteristic curve and decision curve were used to evaluate the accuracy and clinical usefulness of the prediction model.

**Results:**

Positron emission tomography-based local objective response rate was significantly higher than that based on computed tomography [70.97% (22/31) and 12.90% (4/31), respectively (p<0.001)]. The mean survival time of responders and non-responders assessed by positron emission tomography was 28.6 months vs. 11.4 months (p=0.29), whereas that assessed by computed tomography was 24.5 months vs. 26 months (p=0.66), respectively. Three radiomics features were screened to establish a personalized prediction nomogram with high area under curve (0.94, 95% CI 0.85–0.99, p<0.001). The decision curve showed a high clinical value of the radiomics nomogram.

**Conclusions:**

We recommend positron emission tomography for evaluating the short-term efficacy of hypo-fractionated radiotherapy in non-small cell lung cancer, and that the radiomics nomogram could be an important technique for the prediction of short-term efficacy, which might enable an improved and precise treatment.

**Registration number/URL:**

ChiCTR1900027768/http://www.chictr.org.cn/showprojen.aspx?proj=46057

## Introduction

Lung cancer is the most common cancer type and the leading cause of cancer-associated mortality worldwide (1). Hypo-fractionated radiotherapy (HFRT) includes stereotactic body radiation therapy (SBRT) and hypo-fractionated brachytherapy, both of which deliver a high biologically effective dose (BED) to the tumor while minimizing toxicity to the normal tissues. Therefore, HFRT can prompt superior local control and improved survival (2).

Currently, lung cancer treatment efficacy evaluation is mostly based on computed tomography (CT) (3). However, CT being a structural imaging, has limited value in the detection of an early response to therapy, and the tumors could be obscured by atelectasis and radiation pneumonitis (4). Instead, functional imaging with 18F-fludeoxyglucose positron emission tomography (^18^F-FDG PET) may promote accurate and early assessment of therapy response (5–7). In addition, recent studies have demonstrated that radiomics has been successfully used to stage the tumor, assess the side effects, and predict the clinical endpoints in lung cancer (8–10). However, to our knowledge, few studies have focused on contrasting the efficacy of PET and CT in peripheral non-small cell lung cancer (NSCLC) after HFRT treatment as well as on the development of a prediction model of local short-term efficacy based on PET radiomics.

Therefore, the main objectives of this prospective trial were to investigate the differences between short-term efficacy of PET and CT while evaluating the effects of HFRT in peripheral NSCLC, and to screen the efficacy-related radiomics features of PET imaging and use those to establish a prediction model.

## Materials and Methods

### Patients

PET imaging was performed as a part of the phase I/II clinical trial (Clinicaltrials.gov number: ChiCTR1900027768) that evaluated the efficacy of HFRT in patients with pathologically confirmed NSCLC. The patients who underwent HFRT for primary NSCLC (T2-4N0-3M0-1) at our hospital, between March 2015 and June 2019 were enrolled in this study. Eventually, 31 lesions of 30 patients underwent serial 18F-FDG PET/CT 1 month before HFRT and 3 months after HFRT on the same scanner. The TNM stage was designated according to the American Joint Committee on Cancer Staging 8th edition (AJCC) (11). The N and M staging was based on pre-treatment PET/CT and magnetic resonance imaging (MRI). Additionally, all patients underwent pathological diagnosis for the lung lesion before undergoing treatment. The first follow-up imaging examination was performed 4–12 weeks after radiotherapy. After that, patients were monitored every three months in the first year, every six months in the next two years, and once a year thereafter. Because the patients lived in remote places, the scan was occasionally performed outside these limits. The study was approved by the Research Ethics Committee of the Affiliated Hospital of Southwest Medical University (Date of approval by ethics committee and approval number: 2013-8-26, and KY2019276, respectively) and conducted in accordance with the Declaration of Helsinki (as revised in 2013) and its later amendments or comparable ethical standards. Written informed consent was obtained from all individual participants included in the study.

### Treatment

All patients were treated with radiotherapy planning system (TPS) (Oncentra 4.3, Elekta, Sweden). Then, 19 out of 30 patients were treated with hypo-fractionated brachytherapy, delivered with an ^192^Ir source from a MicroSelectron afterloader (Elekta Brachytherapy, Elekta AB, Stockholm, Sweden), and these patients were administered a single dose of 30Gy, as recommended by a previous clinical trial (12). The remaining 11 patients were treated with SBRT that was delivered in 3–5 fractions to a total of 23–50Gy. After radiotherapy, concurrent platinum-based doublet adjuvant chemotherapy was allowed. In addition to cisplatin (or carboplatin), a second concurrent nonplatinum agent was required (e.g., paclitaxel, etoposide).

### PET and CT Scanning Acquisition and Processing

Patients fasted for at least 6 h before ^18^F-FDG was administered. The patient’s blood glucose level should have been ≤ 11 mmol/L. The PET-CT was performed according to the European Association of Nuclear Medicine (EANM) guidelines version 1.0 (13). A whole-body PET-CT (Philips Gemini TF/16; Philips, Cleveland, Ohio, USA) was performed after the intravenous administration of ^18^F-FDG (5.55 MB q/kg). Then, low-dose helical CT transmission scanning (pitch, 0.813; current, 100 mAs; peak voltage, 120 kV; slice thickness, 5.0 mm) was performed with attenuation correction and lesion localization. PET was then performed at 1.5 min per bed position and used 19–21 bed positions. ^18^F-PET/CT scanning was performed from the vertex of the head to the feet. In order to reduce the impact of respiratory motion on image acquisition and ensure the credibility of the research results, chest scans were conducted after having the patients hold their breath.

### Assessment of Local Objective Response Rate

The PET-only and CT-only images were both sent to the three-dimensional (3D) radiotherapy planning system (TPS) (Oncentra 4.3, Elekta, Sweden) *via* the local area network. One physician with more than 15 years of experience with PET and CT in peripheral lung cancer and regions of interest (ROI) definition performed the analyses after being blinded to the patient outcome data. Nodal disease was not included in the analysis. PET-based ROI delineation was carried out before and after HFRT with standard uptake value (SUV) of 2.5 as the initial threshold (14, 15). Mean standard uptake value (SUVmean), maximum standard uptake value (SUVmax), metabolic tumor volume (MTV), and longest diameter (Dmax) were calculated for the ROI based on PET. CT-based ROI was manually drawn by the same physician with lung windows (window width, 1600 Hounsfield units [HU]; window level, 600 HU). Volume and longest diameter from the ROI based on CT were also calculated. CT-only scans were assessed for response using Response evaluation criteria in solid tumors (RECIST1.1) (16) after the treatment, and PET-based response criteria used in this study were according to the European Organization for Research and Treatment of Cancer (EORTC) (17) that were based on an assessment of the SUVmax measured through ROI analysis. The terms for the response categories were the same for both CT and PET: complete response (CR), partial response (PR), stable disease (SD), and progressive disease (PD).

### Radiomic Processing

The workflow for radiomic processing included the following four steps: image acquisition and reconstruction, image segmentation, feature extraction, and data analysis (18, 19). The first two steps involving collection of PET images and delineation of the ROI were described in the above section. The feature extraction and definition in this study were consistent with the Imaging Biomarker Standardization Initiative (IBSI) (20). The feature extraction process was divided into image processing and feature calculation. For each ROI based on PET image, a resampled 4×4×4 mm^3^ voxel size and a bin width of 25 were applied (21). After image processing, Due to the characteristics of medical images, filter properties are important for image analysis methods. Filtering properties including geometric invariances for medical image analysis directional sensitivity, combining directional sensitivity and invariance to local rotations, spectral coverage (22). We used wavelet which is a filtering method based on a collection with eight combinations of applying either a high or a low pass filter and cover the entire image spectrum as study filter. Wavelets families contains the following wavelet groups: “haar,” “db,” “sym,” “coif,” “bior,” “rbio,” “dmey” (22). After those, all feature classes with the exception of shape can be calculated on the original image and a derived image which obtained by applying wavelets filter. Feature extraction was based on the 3Dslicer platform and used the pyradiomics package, which is available at: http://PyRadiomics.readthedocs.io/en/latest/, accessed June 30, 2019 (23).

### Statistical Analysis

We assessed the differences between all parameters observed by CT and PET using the Wilcoxon signed-rank test or paired t test after ascertaining whether the parameters were normally distributed using the Shapiro-Wilks test. The delta-parameters of the CT and PET were identified by the prefix “Δ.”

Tumor response to HFRT was analyzed using PET and CT independently. Patients were then grouped as responders (CR+PR) or non-responders (SD+PD). The difference of ORR between PET and CT was assessed using the Mann-Whitney U test. The Kaplan Meier method was used to analyze the local objective response and subsequent survival duration of the two imaging methods.

The least absolute shrinkage and selection operator (LASSO) regression analysis was used to screen out the radiomics features related to responders, and logistic regression analysis was used to develop the curative-effect-predicting model. To assess the probability of short-term efficacy in individuals, we built the radiomics nomogram based on multivariable logistic analysis. Receiver operating characteristic (ROC) curve and area under the curve (AUC) were used to assess the accuracy of the prediction model. To ensure radiomic robustness, we performed 1,000 bootstrap resamples to check the agreement between our prediction based on radiomics and actual observation. The net benefit was quantified by the decision curve analysis to determine the clinical applicability of the PET-based radiomics nomogram prediction model.

All statistical analyses were performed using SPSS, Version 17.0 software (SPSS, Inc., Chicago, IL) and R software, version 3.6.3 (R Foundation for Statistical Computing, Vienna, Austria), using the glmnet, rms, and pROC analysis packages for Windows. The level of statistical significance was defined as a p value less than 0.05 based on 2-sided tests.

## Results

### Baseline Characteristics and Follow-Up

From March 2015 to June 2019, 30 patients with peripheral NSCLC were enrolled. The baseline clinical characteristics of these patients are listed in **Tables 1** and **2**.

**Table 1:** Characteristics of the patients.

Characteristics	Median (Range) or Number (%)
Age (y)	55.5 (43–77)
Gender	
Male	24 (80.00)
Female	6 (20.00)
Stage	
IIIA~IIIC	8 (26.67)
IVA~IVB	22 (73.33)
Histology	
Adenocarcinoma	22 (73.33)
Squamous carcinoma	8 (26.67)
Smoking	
Yes	18 (60.00)
No	12 (40.00)
KPS	
70~80	15 (50.00)
90~100	15 (50.00)
ECOG	
0	15 (50.00)
1~2	15 (50.00)
Location	
Left	13 (41.94)
Right	18 (58.06)
Radiotherapy	
Stereotactic body radiation therapy	12 (38.71)
Hypo-fractionated brachytherapy	19 (61.29)

KPS, Karnofsky Performance Status; ECOG, Eastern Cooperative Oncology Group.

**Table 2:** Patient TNM staging, duration and type of chemotherapy, and duration of response.

Patient no.	Stage	Type of chemotherapy	Chemotherapy cycle	Duration of response (month)
1	T3N0M1	Pemetrexed+cisplatin	4	3
2	T4N2M0	Etoposide+ cisplatin	3	6
3	T3N2M0	None	\	3
4	T4N0M1	None	\	3
5	T4N2M1	None	\	3
6	T4N3M1	None	\	1
7	T4N2M1	None	\	3
8	T2N3M0	Paclitaxel+ carboplatin	2	3
9	T4N3M0	Etoposide+ cisplatin	4	6
10	T2N2M0	None	\	6
11	T4N2M1	None	\	6
12	T4N3M1	Etoposide+ cisplatin	3	6
13	T4N2M1	Paclitaxel+ carboplatin	1	1
14	T4N2M1	Paclitaxel+ carboplatin	1	1
15	T4N2M1	Paclitaxel+ carboplatin	1	3
16	T4N2M0	None	\	6
17	T2N3M0	None	\	1
18	T4N1M1	None	\	3
19	T4N3M1	None	\	1
20	T3N1M1	None	\	1
21	T3N1M1	None	\	3
22	T4N0M0	None	\	3
23	T3N3M1	None	\	6
24	T2N1M1	None	\	3
25	T4N3M1	None	\	3
26	T4N3M1	None	\	3
27	T4N3M1	None	\	6
28	T1N2M1	Etoposide+carboplatin	3	3
29	T4N2M1	None	\	1
30	T2N1M1	None	\	1

Survival was measured from the date of completing radiotherapy to the date of death from any cause since April 2020. Patients who were still alive at the date of last contact were censored at deadline. Only one patient was lost to follow-up before the deadline date; nine patients died and the rest were still alive. Estimated median follow-up duration was 16 months.

### Comparison of PET- and CT-based Response Assessments

**Figure 1:**
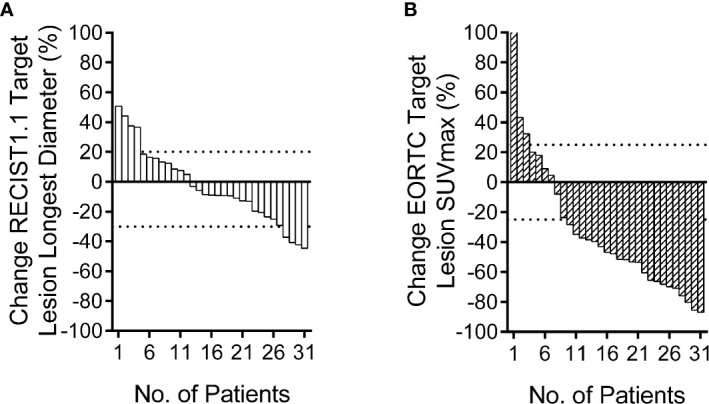
Relative change rate of longest diameter and maximum standard uptake value (SUVmax) in each patient. Best overall response waterfall plots, in which computed tomography (CT) **(A)** is based on the rate of longest diameter changes, according to RECIST 1.1, and positron emission tomography (PET) **(B)** is based on the rate of SUVmax changes, according to European Organization for Research and Treatment of Cancer (EORTC). The top dotted line represents progressive disease, the bottom dotted line represents partial response, or complete response, while, stable disease is represented by the area between the two dotted lines.

**Figure 2:**
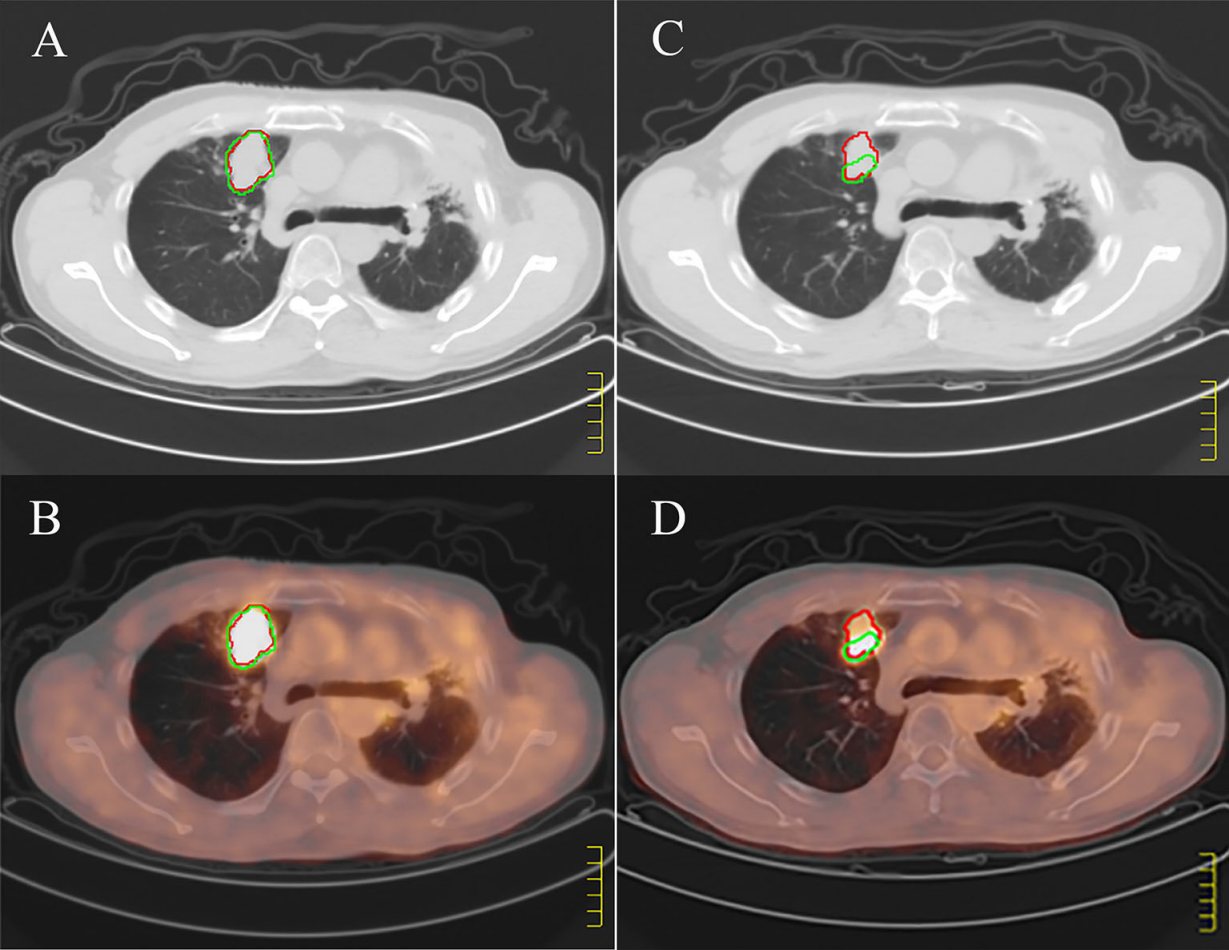
Example of discordant positron emission tomography (PET/CT) and computed tomography (CT). **(A, B)** show pretreatment CT and PET/CT images respectively, and **(C, D)** show CT and PET/CT images of the large tumors 3 months after treatment. The red regions of interest (ROI) is manually segmented based on CT, and the green ROI is delineated according to SUV value higher than 2.5 based on PET. According to the images before and after treatment, CT shows stable disease; PET/CT shows partial response.

The PET based efficacy in all 31 lesions was assessed as CR (n=0), PR (n=22), SD (n=6), and PD (n=3), while the same according to CT was CR (n=0), PR (n=4), SD (n=23) and PD (n=4). Comparisons for all patients are shown in **Figure 1**. For these patients assessable by both PET and CT, there was a poor agreement between the two assessments, with a weighted kappa value of 0.114. A significantly higher number of patients were regarded as responders on PET than on CT in the patients assessable by both techniques (22 vs. four patients respectively; p<0.001). All differences between parameters observed by CT and PET are listed in **Table 3**. A representative case of an individual patient exemplifying the different CT and PET/CT response is shown in **Figure 2**.

**Table 3:** Comparison of difference variables and short-term efficacy assessment based on PET and CT.

Variable	PET/CT	CT	p Value
Tumor volume (cm³)			
V_B_	25.31 (14.92,89.79)	28.54 (15.05,76.95)	0.337^†^
V_L_	6.40 (2.14,14.46)	18.25 (8.59,67.02)	<.001^†^
ΔV	−18.07 (−63.04, −8.58)	−13.93 (−49.56, 5.03)	<.001^†^
D-max (cm)			
D_B_	5.19 (3.53,6.80)	5.30 (3.99,7.38)	0.019^†^
D_L_	3.19 (1.62,3.93)	4.59 (3.83,7.83)	<.001^†^
ΔD	−1.66 (−3.81, −0.73)	−0.35 ± 1.30	<.001^†^
SUVmean_B_	4.78 (3.92,5.47)	/	/
SUVmax_B_	9.92 (7.52,11.77)	/	/
SUVmax_L_	4.72 (3.31,7.83)	/	/
ΔSUVmax	−4.17 ± 5.25	/	/
Efficacy^§^			<.001^‡^
Responders	22 (70.97%)	4 (12.90%)	
Non-responders	9 (29.03%)	27 (87.10%)	

PET, positron emission tomography/computed tomography; CT, computed tomography; the suffix B, value of volume/longest diameter before brachytherapy; the suffix L, value of volume/longest diameter after hypo-fractionated brachytherapy (HFBT). Use mean ± SD for normally distributed data and interquartile ranges (IQRs) for data that are not normally distributed.

ΔV=V_L_– V_B_, ΔD=D_L_ – D_B_, ΔSUVmax=SUVmax_L_–SUVmax_B_.

^†^p value of the Wilcoxon signed rank test.

^‡^p value of the McNemar test continuity correction.

^§^PET-based and CT-based evaluation of efficacy is according to the European Organization for Research and Treatment of Cancer (EORTC)and Response Evaluation Criteria in Solid Tumors (RECIST 1.1) respectively; complete response or partial response means responders, progressive disease or stable disease means non-responders.

### Effect of Chemotherapy on Treatment Evaluation

**Figure 3:**
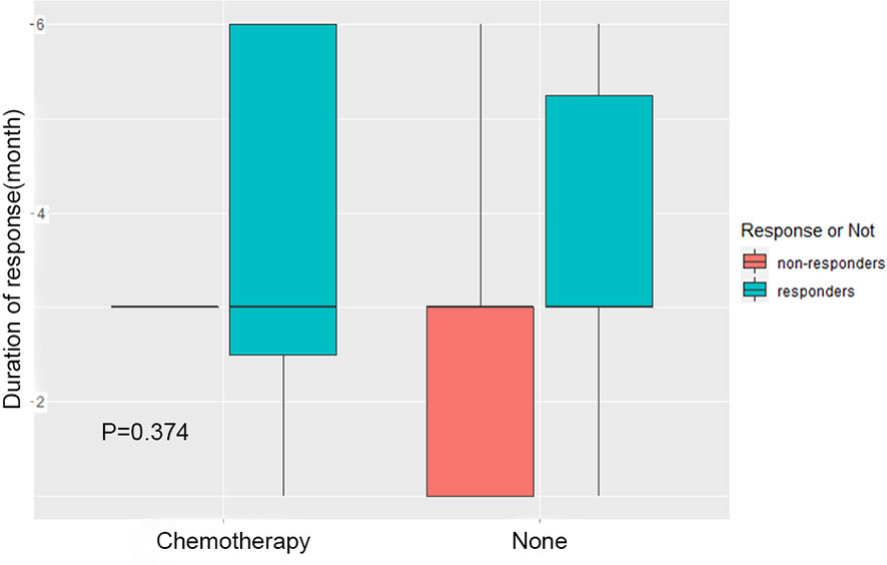
Effect of chemotherapy on treatment evaluation. The patients were divided into two groups: chemotherapy group after radiotherapy (Chemotherapy) and group without chemotherapy after radiotherapy (None). The difference of short-term efficacy between the two groups was compared.

In order to explore the effect of chemotherapy after radiotherapy on the evaluation of short-term efficacy, the patients were divided into two groups: post radiotherapy chemotherapy group (Chemotherapy) and non-chemotherapy treatment group (None). The difference of curative effect between the two groups was compared. The results showed that there was no significant difference between the patients receiving chemotherapy and those who did not receive chemotherapy (**Figure 3**, p=0.374), that is, no matter whether chemotherapy was carried out after radiotherapy, the short-term efficacy evaluation of patients had no influence.

### Prognostic Significance of Response Assessments

**Figure 4:**
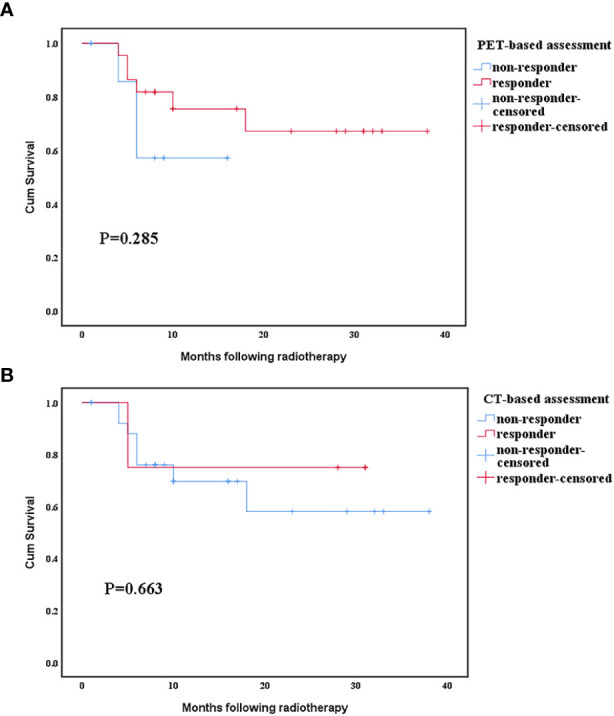
Relationship between survival time and response evaluated by positron emission tomography (PET) and computed tomography (CT) scan. **(A)** Comparison of positron emission tomography scan response categories. **(B)** Comparison of computed tomography scan response categories.

The mean survival time of responders and non-responders assessed by PET was 28.6 months vs. 11.4 months, whereas that assessed by CT was 24.5 months vs. 26 months, respectively. In PET assessment, the survival duration of responders was longer than that of non-responders, whereas CT assessment had the opposite result. Thus, the PET responses seem to be stronger prognostic indicators than the CT responses. However, neither the PET nor CT scan assessments of response had a significant difference in subsequent survival duration between the responders and non-responders (**Figures 4A, B**).

### Radiomics Feature Selection and Response Prediction

**Figure 5:**
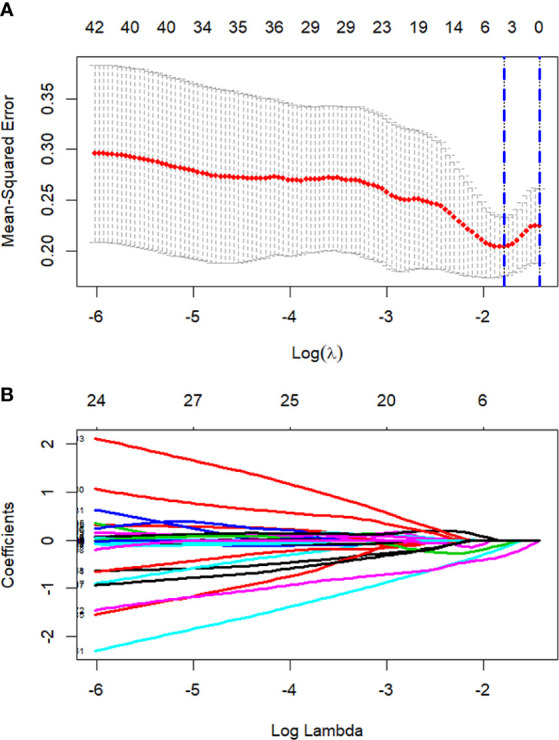
Process of LASSO regression screening of radiomics features. Screening radiomics features using the least absolute shrinkage and selection operator (LASSO) binary logistic regression model. **(A)** Tuning parameter (λ) in the LASSO model used 5-fold cross-validation. Dotted vertical lines are drawn by using the minimum criteria and the 1 standard error of the minimum criteria. **(B)** 851 normalized lasso coefficient plots of radiomics features. When log (λ) takes the minimum criteria, three non-zero coefficients are selected.

**Figure 6:**
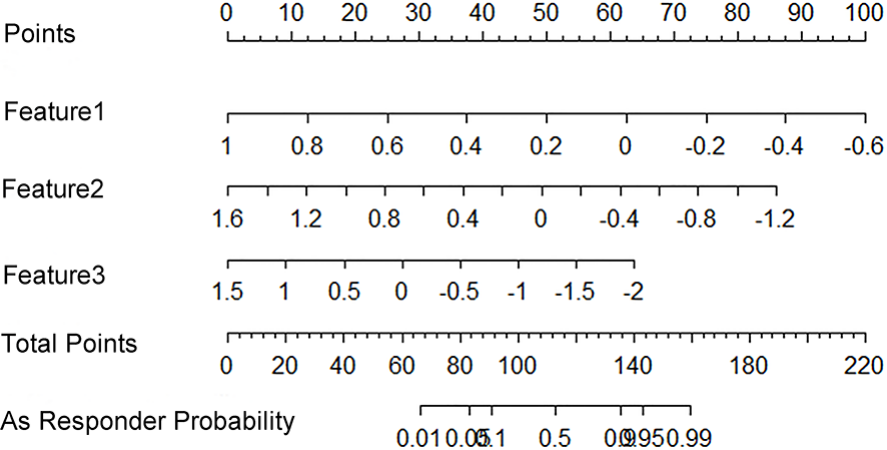
A radiomics nomogram that predicts the probability of effective treatment. Feature1: Wavelet.LHL_NGTDM_Busyness; Feature2: Wavelet.LHH_GLRLM_ShortRunHighGrayLevelEmphasis; Feature3: Wavelet.HHH_Firstorder_Median.

**Figure 7:**
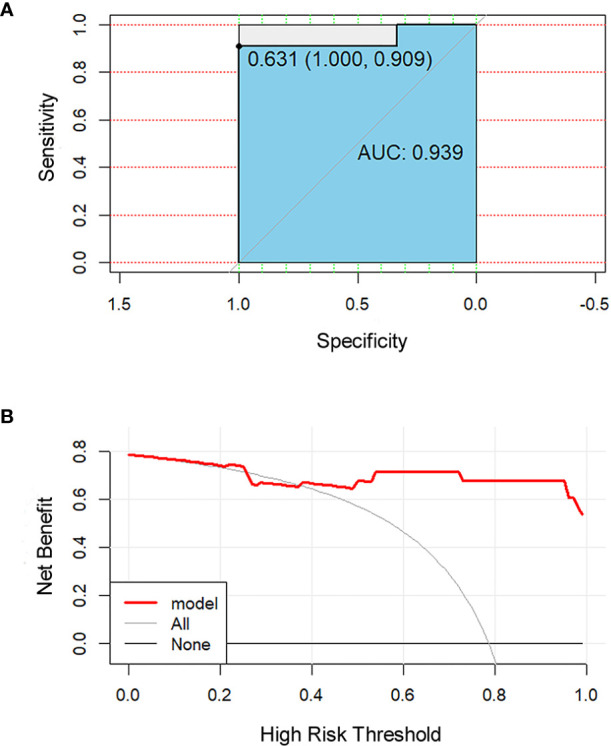
Receiver operating characteristic (ROC) and decision curve for the model. The ROC curve **(A)** shows the prediction accuracy of the model (AUC 0.94, 95% CI 0.85–0.99, p < 0.001). In the decision curve **(B)**, the red line represents the radiomics nomogram. The gray line represents the assumption that all patients were responders. The thin black line represents the assumption that all patients were non-responders.

All 851 radiomics features were extracted, including Shape features, First Order statistical features, Gray Level Co-occurrence Matrix (GLCM) features, Gray Level Dependence Matrix (GLDM) features, Gray Level Run Length Matrix (GLRLM) features, Gray Level Size Zone Matrix (GLSZM) features, and Neighboring Gray Tone Difference Matrix (NGTDM) features. Among the radiological features, 851 features were reduced to three potential predictors based on 31 lesions (**Figure 5**), which were Busyness of NGTDM of wavelet-LHL, Short Run High Gray Level Emphasis of GLRLM of wavelet-LHH, and Median of First order of wavelet-HHH. These three features were used to establish a model that is presented as a nomogram in **Figure 6**. Based on the ROC curve analysis, the model with high AUC (0.94, 95% CI 0.85–0.99, p<0.001) is presented in **Figure 7A**, and the decision curve analysis for the model is presented in **Figure 7B**. The decision curve indicates that if the threshold probability of a patient is 40%, then the use of radiographs from the current study to predict treatment outcomes would add more benefits than the “treat-all-patients” or “treat-none” options.

## Discussion

Currently, CT is the recommended method for response assessment in NSCLC as per the RECIST guidelines. There is insufficient standardization or evidence to abandon anatomical assessment of tumor burden, or to determine if it is appropriate to replace the unidimensional anatomic assessment with either volumetric anatomical assessment or functional assessment with PET (16). Some studies have revealed that PET has better evaluation validity than CT in patients with both NSCLC (7) and small cell lung cancer (24). Our data revealed that among such patients, who are assessable by both techniques, more patients were regarded as responders on PET than on CT. The local ORR of patients according to PET was 70.97%, whereas the corresponding CT evaluation found that to be 12.90%. Moreover, when the efficacy evaluation was compared between CT and PET, only eight lesions (25.81%) were evaluated uniformly, of which, four were evaluated as PR and four were evaluated as SD. Some researchers have previously reported that PET/CT-derived tumor volumes were smaller than those derived by CT alone in case of locally advanced-stage peripheral lung cancer before radiotherapy treatment (25). However, we found that the volumes of ROIs, delineated by PET and CT before HFRT, were similar. In contrast, Dmax of ROIs delineated using PET before and after HFRT, and the volumes of ROIs after HFRT delineated using PET were significantly smaller than that of ROIs outlined using CT, as shown in **Table 3**, mainly because CT was not very sensitive to distinguish between atelectasis and lung cancer. Moreover, radiation therapy may result in radiation-induced lung opacity (RILO) on CT (5), such as ground-glass opacity, scar or fibrotic changes, consolidation with air-bronchogram, consolidation alone, and nodule. These RILOs may result in a larger size and diameter of the tumor being observed on the CT, which may overestimate the progressive disease during exclusive assessment of efficacy *via* the CT. A previous study had also suggested that the combined PET/CT to evaluate post-treatment response would increase the correct identification of patients with progressive disease after lung SBRT (26). While investigating the differences in the PET and CT based response assessment, we also report significant statistical differences between the two methods. As shown in **Figure 1**, only six patients were considered SD based on PET, whereas 23 patients were considered SD based on CT. According to our study, PET assessment is better than CT assessment in reflecting the prognosis of patients. Although there was no significant difference in the survival time between the two groups of patients, this may be due to the small sample size or short follow-up time. Based on the above data, we recommend that PET would be better than CT when evaluating the efficacy of HFRT in NSCLC, since the change in tumor volume may be slower than the metabolic change discernable by PET; additionally, CT scan may not accurately reflect the therapeutic effect on the tumor in time, which may lead to unnecessary overtreatment.

Since each patient’s sensitivity to treatment is inconsistent, the prognosis may be completely different even in patients who are in the same cancer stage and receive the same treatment (27). Therefore, early prediction of treatment response is particularly important for identifying patients who may or may not benefit from treatment.

FDG uptake is not only related to increased metabolism, but also to other physiological parameters, such as cell proliferation (28), perfusion, invasiveness, and hypoxia (29). Therefore, radiomics can obtain several data contained in the PET image through non-invasive means. Many quantitative features of PET can be calculated during treatment of the patient. This principle of extracting image features is termed as “radiomics” that has been recently studied in esophageal cancer (30), NSCLC (31, 32), breast cancer (33), nasopharyngeal carcinoma (34), and rectal cancer (35), and demonstrated its potential in predicting treatment efficacy or patient prognosis.

PET-based radiomics had a high sensitivity in AUC for predicting the efficacy of radiochemotherapy in esophageal cancer (76%–92%) (30). In the prediction of the efficacy of adjuvant therapy for rectal cancer, the AUC of radiomics based on PET and MRI was up to 0.86 (35). Our model based on PET radiomics has an AUC of 0.94, indicating that PET radiomics plays a significant role in predicting the treatment efficacy in non-small cell lung cancer. The most important use of nomograms is based on explaining the individual’s need for further treatment. Therefore, in order to prove its clinical value, we evaluated whether the radiomics nomogram assisted decision-making could add more benefits to the patients through the novel method of decision curve, which estimated the net benefit. (Net benefit was defined as the proportion of true positives minus the proportion of false positives, weighted by the relative harm of false-positive and false-negative results) (36).

The key to ensure the accuracy, generalization and repeatability of radiomics prediction is accurate and high repeatability ROI segmentation. In this study, all ROI are manually segmented. However, manual segmentation has the disadvantages of time-consuming and low repeatability of tumor volume description. Automatic or semi-automatic methods can make up for these defects. Recently, a lot of research on automatic segmentation is increasing rapidly. The fully automatic multi-mode PET/MRI segmentation method proposed by some scholars is an operator independent method, which can help clinicians to outline containing both metabolic and morphological information (37). Some studies have shown that, compared with traditional manual segmentation radiomics approaches, the survival model of automatic tumor segmentation based on neural network segmentation shows significantly higher predictive power (38). However, there is not enough evidence to prove that automatic segmentation can replace manual segmentation. We have only preliminarily discussed the prediction performance of manual segmentation. We will test accurate automatic segmentation methods for reliable segmentation.

## Limitations and Conclusion

First, this study is based on a small-sample clinical trial to establish a training model. For the training sample size, some scholars suggest that for multiple regression, each prediction variable needs at least 10 observations to produce a reasonable and stable estimate (39). In our study, three features are selected as the final model, and the minimum data size is 30. Due to the limited sample size, in order to make the model more accurate, we use all the collected cases to establish the training set. Like the previous small sample study (40), this study uses bootstrap resampling method to extract multiple samples from the original samples, generate simulation data, and compare these data results with the actual results to prove the robustness of the statistical data. In order to better understand whether the different short-term efficacy evaluations obtained by the two imaging techniques are related to the patient’s long-term prognosis, we plan to increase the follow-up time to calculate the 3- or 5-year overall survival rates, and respective disease-free survival rates. Finally, our model needs to be validated through further prospective research, although it showed a high predictive power. Though we have accord with the requirement of the minimum sample size in training set and used LASSO regression to avoid overfitting (41), we still need to increase the sample size and set the training set to further avoid overfitting. We also plan to include more patients in further prospective studies, wherein some patients will continue to serve as the training set to increase the repeatability of the prediction performance of the model by expanding the sample size, while others will serve as the validation set to verify the accuracy of the verification model. The study provides further evidence to use PET to evaluate the efficacy in NSCLC. Our results show that early ^18^F-FDG-PET could be particularly useful for identifying early responders, allowing clinicians to avoid overtreatment, and that the radiomics nomogram could be an important technique for the prediction of short-term efficacy in patients with NSCLC, which might enable an improved and precise treatment (41).

## Data Availability Statement

The original contributions presented in the study are included in the article/**Supplementary Material**. Further inquiries can be directed to the corresponding authors.

## Ethics Statement

The studies involving human participants were reviewed and approved by the Affiliated Hospital of Southwest Medical University Clinical trial ethics committee. The patients/participants provided their written informed consent to participate in this study.

## Author Contributions

Conception and design: SL, H-WP, and Y-QJ. Administrative support: SL and J-BW. Provision of study materials or patients: YC and YZ. Collection and assembly of data: HC, X-XS, and QG. Data analysis and interpretation: Y-QJ and HC. Manuscript writing: Y-QJ and H-WP. Final approval of manuscript: SL. All authors contributed to the article and approved the submitted version

## Funding

This work was supported by the grants from the National Natural Science Foundation of China (no. 81201682), the Scientific Research Foundation of the Luzhou Science and Technology Bureau (no. 2016LZXNYD-J05), and the Southwest Medical University Foundation (no. 201617).

## Conflict of Interest

The authors declare that the research was conducted in the absence of any commercial or financial relationships that could be construed as a potential conflict of interest.
