# Supplementary material for: Positron Emission Tomography-Based Short-Term Efficacy Evaluation and Prediction in Patients With Non-Small Cell Lung Cancer Treated With Hypo-Fractionated Radiotherapy
Source: Front Oncol. 2021 Feb 25;11:590836. doi: 10.3389/fonc.2021.590836 (PMC7947869; doi:10.3389/fonc.2021.590836)
Supplement: Supplementary file 1 [file DataSheet_1.zip › data/detailed statement on the data and code.docx]

1、First of all, the meaning of each file is introduced. The Microsoft Excel named “bra+SBRT+clin+radi-0” represents all the original data, while “data4” is derived from “bra+SBRT+clin+radi-0” after code processing, and ”box-and-whisker plot” is sorted out from “bra+SBRT+clin+radi-0” according to the box-and-whisker plot.

2、“R-Code “ is the code for R analysis. It is mainly divided into the following steps(“#”represents each step, and the number of “#” is random and does not represent other meanings):

1) Environmental preparation

2) load package

3) Import data

4) Data standardization

5) Lasso regression: a. Convert matrix to data frame. b. Set the number of seeds (to ensure consistent running results). c. 5-fold cross validation. d. Obtain the coefficients of target features.

6) Multi parameter logistic regression

7) The extracted target features are sorted into a new Microsoft Excel "data4", and the data4 is imported

8) View outliers and remove them

9) Repackage the data, construct the logistic model and draw the nomogram

10) Draw the ROC curve of the model

11) The robustness of the model is analyzed

12) Drawing decision curve

3、According to the Microsoft Excel named “bra+SBRT+clin+radi-0”, the patients were divided into four types: responder with chemotherapy, responder without chemotherapy, non-responder with chemotherapy and non-responder without chemotherapy. They are listed in Microsoft Excel named ”box-and-whisker plot”.

4、“box-and-whisker plot“ code is based on Microsoft Excel named ”box-and-whisker plot” for R analysis.
